# Rapid regulation of protein activity in fission yeast

**DOI:** 10.1186/1471-2121-9-23

**Published:** 2008-05-05

**Authors:** Cathrine A Bøe, Ignacio Garcia, Chen-Chun Pai, Jeffrey R Sharom, Henriette C Skjølberg, Erik Boye, Stephen Kearsey, Stuart A MacNeill, Michael D Tyers, Beáta Grallert

**Affiliations:** 1 Department of Cell Biology, Rikshospitalet-Radiumhospitalet Medical Centre, Montebello, 0310 Oslo, Norway; 2 Department of Zoology, University of Oxford, South Parks Road, Oxford OX1 3PS, UK; 3 Department of Molecular Biology, University of Copenhagen, Københavns Biocenter, Ole Maaløes Vej 5, 2200 Copenhagen N, Denmark; 4 Samuel Lunenfeld Research Institute, Room 1080, Mount Sinai Hospital, 600 University Avenue, Toronto Ontario, M5G 1X5, Canada

## Abstract

**Background:**

The fission yeast *Schizosaccharomyces pombe *is widely-used as a model organism for the study of a broad range of eukaryotic cellular processes such as cell cycle, genome stability and cell morphology. Despite the availability of extensive set of genetic, molecular biological, biochemical and cell biological tools for analysis of protein function in fission yeast, studies are often hampered by the lack of an effective method allowing for the rapid regulation of protein level or protein activity.

**Results:**

In order to be able to regulate protein function, we have made use of a previous finding that the hormone binding domain of steroid receptors can be used as a regulatory cassette to subject the activity of heterologous proteins to hormonal regulation. The approach is based on fusing the protein of interest to the hormone binding domain (HBD) of the estrogen receptor (ER). The HBD tag will attract the Hsp90 complex, which can render the fusion protein inactive. Upon addition of estradiol the protein is quickly released from the Hsp90 complex and thereby activated. We have tagged and characterised the induction of activity of four different HBD-tagged proteins. Here we show that the tag provided the means to effectively regulate the activity of two of these proteins.

**Conclusion:**

The estradiol-regulatable hormone binding domain provides a means to regulate the function of some, though not all, fission yeast proteins. This system may result in very quick and reversible activation of the protein of interest. Therefore it will be a powerful tool and it will open experimental approaches in fission yeast that have previously not been possible. Since fission yeast is a widely-used model organism, this will be valuable in many areas of research.

## Background

Regulating protein function or protein level is often useful in order to investigate diverse biological processes. The fission yeast *Schizosaccharomyces pombe *is a popular model organism. It is genetically tractable and a wide variety of methods have been developed to facilitate molecular genetic manipulations in *S. pombe*.

It is usually more advantageous to regulate the activity of the target protein than the protein level, because this results in faster regulation of the protein's activity at wild type protein levels. The most commonly used approach to regulate the activity of the protein of interest is the isolation of conditional mutants, which have been vital tools in many areas of research. Indeed, one of the many advantages of fission yeast as a model system is that it is haploid, which makes it easier to isolate and work with conditional mutants. Most conditional mutants are temperature sensitive. However, not all genes can be mutated such that the corresponding protein becomes temperature sensitive. Furthermore, a temperature shift in itself might stress the cells. Temperature-sensitive proteins often have considerable residual activity at the restrictive temperature such that they rescue the temperature-sensitive mutant when overexpressed. Another common problem is that many temperature-sensitive proteins are not fully active at the permissive temperature. Therefore, temperature shifts of temperature-sensitive mutants are frequently far from the ideal "on" and "off" states that might be desired when regulating protein function. The reversibility of the inactivation varies greatly from mutant to mutant. Upon shift back to the permissive temperature, some temperature-sensitive proteins regain their activity, thus allowing block-and-release experiments. However, many other temperature sensitive proteins do not regain their activities after a period of temperature shift or are degraded at the restrictive temperature. Temperature-sensitive mutants have been particularly useful to explore the functions of essential proteins. However, it is difficult to identify temperature-sensitive mutants of non-essential genes, unless their function is known so that appropriate screens can be designed.

Regardless of the many advantages associated with the use of conditional mutants, they are not always available or applicable. A commonly used alternative is regulating the level of the protein of interest, either by regulating transcription or by regulating protein degradation (see below).

Numerous plasmids have been designed for regulated expression of genes [1], but there are no good tight and rapidly inducible promoters for use in fission yeast. The nmt1 (*n*o *m*essage in *t*hiamine) promoter was the first regulatable promoter to be described in fission yeast [2] and it remains the most commonly used one. This promoter is strong, but mutated versions with reduced strengths are available [3]. The promoter is repressed by thiamine (vitamin B1). The main drawback with the *nmt *promoter is that induction of protein expression is rather slow and it takes several generations to achieve full activation, presumably because the cellular vitamin pools have to be depleted first. Furthermore, thiamine confers over 100-fold repression of nmt1-driven transcription, but the promoter is still somewhat leaky and many cloned genes are expressed to near wild-type levels even in the presence of thiamine, such that they can complement chromosomal mutations. Shut-off experiments, where expression of the protein of interest is turned off by the addition of thiamine, are particularly inefficient for stable proteins, since not only is the promoter leaky, but the protein of interest also has to be diluted out as the cells grow.

There are several other and less widely used regulatable promoters that to some extent can be used in fission yeast. Although they confer regulated expression, there are also severe drawbacks to their use, as detailed below. The tetracycline regulatable promoter is a derivative of the Cauliflower Mosaic Virus (CaMV) promoter, fused to a tetracycline binding site [4]. The use of this promoter requires not only cloning the gene of interest behind the CaMV promoter but also manipulating the parent strain such that it expresses the Tet repressor. The *fbp1 *promoter is repressed by glucose but it can only be used in liquid cultures [5]. The invertase promoter is also repressed by glucose and is activated by sucrose within an hour of medium shift. However, the glucose produced by invertase activity leads to repression of the promoter within a short time, so this promoter can only be used for short periods of expression [6]. Since regulation of the latter two promoters requires changing the carbon source, their use implies dramatically changing the growth conditions during the course of the experiment.

Only recently has a uracil-regulateable promoter been described that allows rapid activation and inactivation of transcription [7]. This system is expected to become a useful tool to regulate protein expression, but it should be noted that it might not always be sufficient to regulate transcription levels to achieve efficient regulation of protein levels.

The above regulatable systems all employ heterologous promoters. The expression levels from these promoters might or might not correspond to that from the native promoter of the gene of interest. The degron method, that circumvents this drawback, is based on regulated degradation of the target protein and has been used successfully in fission yeast [8-10]. However, it depends on a temperature shift to 37°C and the degron tag must be on the N-terminus of the target protein. Depending on the stability of the protein of interest, additional measures might also need to be taken to inactivate the protein. One improvement to the method in fission yeast was to combine the degron with an existing temperature sensitive mutation [8,9]. Another strategy that was employed in budding yeast is overexpression of the ubiquitin ligase Ubr1 [10,11]. This approach however cannot be used in fission yeast to improve degron-directed degradation [10]

In summary, despite having a selection of approaches to regulate protein levels, fission yeast researchers often find it difficult to achieve the desired expression level of their favourite proteins.

Here we describe the application of a system that is based on regulated protein function [12,13] without the need for a temperature shift, as opposed to regulated transcription or protein degradation. We have tested the system on four proteins and were able to regulate the activity of two of them.

## Results

### The principle

The approach we have used is based on the normal regulatory activity of the hormone binding domain (HBD) of vertebrate steroid receptors. The Hsp90 molecular chaperone binds the HBD in the absence of estrogen hormones. Upon addition of estradiol a hormone-induced conformational change in the HBD results in the dissociation of Hsp90 [14].

**Figure 1:**
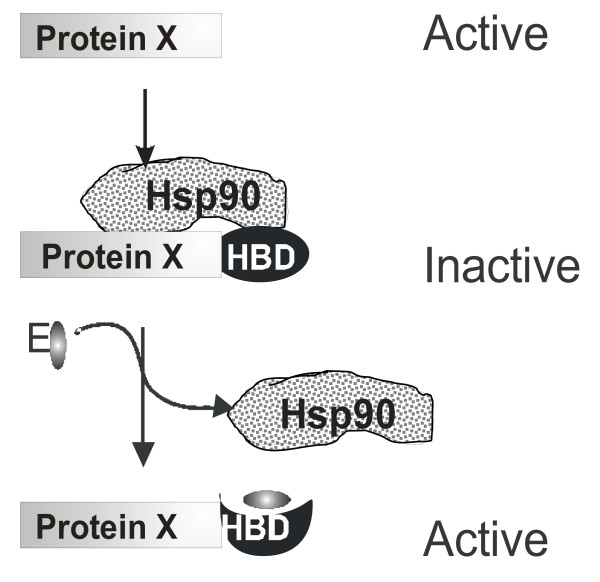
**The principle of regulating protein function by estradiol**. See text for details.

The HBD can also confer sensitivity to estradiol to the activity of heterologous proteins [13]. Fusing a heterologous protein of interest with the hormone-binding domain of the estrogen receptor (ER) renders it inactive presumably because it is bound by the Hsp90 (Fig. 1). Within a few minutes of addition of estradiol the hormone-induced conformational change in the HBD results in dissociation of the Hsp90 and activation of the chimeric protein (Fig. 1) [12,13]. The mechanism of inhibition by Hsp90 is thought to be by steric interference [14] but regulation of the intracellular localization of the chimeric protein has also been reported [15].

In the following sections, we shall refer to the fusion protein as "*active*" or "*inactive*" in quotation marks, reflecting the presence or absence of estradiol, respectively. This indicates the protein activity expected based on the model described above and shown in Figure 1, rather than that observed experimentally.

### Cdc13-des2-HBD

Cdc13 is the mitotic B-type cyclin in fission yeast. Cdc13 protein levels are stringently regulated through the cell cycle. The protein starts accumulating at the G1-S transition until, in late G2, the high level required for entry into and progression through mitosis is reached [16]. Cdc13 is then degraded via the APC (anaphase promoting complex) at the end of mitosis [17,18]. We wished to be able to regulate the Cdc13 levels independently of the cell cycle stage, i.e. allowing regulation that would be independent of APC activity. Therefore we employed a non-degradable mutant form of Cdc13, Cdc13-des2, which lacks the recognition sequence that targets the protein for ubiquitylation by the APC [18].

**Figure 2:**
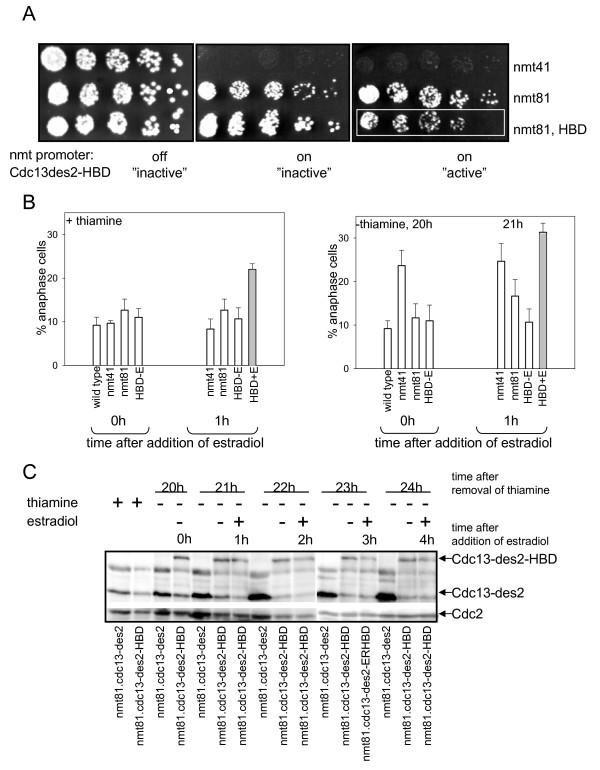
**The activity of Cdc13-des2-HBD is regulated by estradiol**. **A**, Cells transformed with plasmids carrying the nmt.cdc13-des2 and nmt.cdc13-des2-HBD constructs were serially diluted (4X) and plated onto minimal plates with and without thiamine and estradiol as indicated. The cells carrying the ERHBD tagged cdc13-des2 growing poorly in the presence of estradiol are highlighted with a white rectangle. **B**, Expression of Cdc13-des-HBD results in anaphase delay in the presence of estradiol. The nmt promoter was induced for 20 h before addition of estradiol for 1 h. Anaphase index is shown before and 1 h after addition of estradiol. Bars show anaphase indices in the presence (left panel) and absence (right panel) of thiamine. Anaphase index observed in wild type cells is shown for comparison. The bar representing the tagged construct in the presence of estradiol is shaded. **C**, Cdc13 levels are not increased by the presence of the tag or estradiol. Cells carrying the nmt81.cdc13-des2 and nmt81.cdc13-des2-HBD plasmids were grown in minimal medium in the presence of thiamine, then thiamine was washed out to induce the nmt promoter. Estradiol was added to half of the cultures after 20 h induction. Samples for protein extracts were taken at the indicated times. TCA extracts were made and western blot analysis was performed using the SP4 anti-Cdc13 antibody [40] and the anti-PSTAIRE (Santa Cruz) antibody to detect Cdc2 which serves as loading control.

We fused sequences encoding the ER hormone binding domain to the 3' end of the *cdc13-des2 *ORF. It had been previously shown that fission yeast cells expressing Cdc13-des2 from the medium strength nmt41 promoter are inviable when the promoter is induced [18] (Fig. 2A), but the cells are viable when the promoter is repressed. To ensure more physiological levels of Cdc13, we used the weak nmt81 promoter to regulate the expression of the Cdc13-des2-HBD fusion protein.

Expression of Cdc13-des2 or Cdc13-des2-ERHBD from the nmt81 promoter was not lethal even when the promoter was induced (Fig. 2A). However, when estradiol was added (fusion protein "active"), the cells expressing ERHBD-tagged Cdc13-des2 grew very poorly as shown by a spot test of serially diluted cells (Fig. 2A, compare "active" to "inactive"). These observations suggest that the fusion protein is indeed activated in the presence of estradiol.

Fission yeast cells expressing Cdc13-des2 from the medium strength nmt41 promoter delay at the anaphase-telophase transition [18]. To measure more accurately the activity of the Cdc13-des2-HBD fusion protein, we counted anaphase indices in the presence and absence of estradiol and/or thiamine (Fig. 2B). Expression of Cdc13-des2 from the weak nmt81 promoter leads to a marginal increase of anaphase index, whereas expression from the medium strength nmt41 promoter brings about a pronounced anaphase delay (Fig. 2B, white bars). Interestingly, addition of estradiol to cells expressing Cdc13-des2-HBD (fusion protein "active") (Fig. 2B, shaded bar) results in an anaphase delay comparable to that in cells expressing the protein without the HBD tag from the medium strength nmt41 promoter. The anaphase index significantly increases by an hour after hormone addition, indicating a quick response, and remains high for at least one generation time (data not shown). At later timepoints cut cells were observed both with and without the ERHBD tag (data not shown). These data strongly suggest that estradiol indeed activates the Cdc13-des2-HBD fusion protein.

It is noteworthy that expression of Cdc13-des2-HBD produces a higher anaphase index and, at later timepoints after hormone addition, more cut and septated cells than expression of Cdc13-des2 from the same promoter. One possible explanation is that the expression level of Cdc13 and/or the copy number of the plasmid is affected by the presence of estradiol. However, western blot analysis of Cdc13 levels shows no increase of Cdc13 level by the presence of the hormone, nor does the tag increase the amount of the protein (Fig. 2C). We do not observe an increased amount of the endogenous Cdc13 either (Fig. 2C), which would be expected if the HBD tag was cleaved off. If there is a difference, it is that the tagged protein is present in somewhat lower amounts then the untagged protein. We considered the possibility that the HBD tag itself is responsible for the mitotic defects but we deem this most unlikely. The differences between the effects of expressing Cdc13-des2 with and without the HBD tag are quantitative, not qualitative, indicating that the tag itself does not confer a novel function on the fusion protein. Consistently, in the absence of estradiol the cells carrying the tagged construct grow like wild type cells (Fig. 2B). It is likely that a sudden increase of Cdc13 levels upon hormone addition disturbs the localization and/or function of Cdc13 and thus aggravates the effects of overexpressing a non-degradable Cdc13.

A major limitation with the use of the nmt promoter is the high background expression level even in the presence of thiamine. We wished to evaluate the effectiveness of inhibiting Cdc13-des2-HBD protein function with the HBD tag in the absence of estradiol versus repressing expression of Cdc13-des2-HBD from the nmt81 promoter in pREP82 by addition of thiamine to the growth medium. To this end we compared the anaphase indices of cells where we inhibited Cdc13-des2-HBD protein activity by not adding estradiol (but maintained full expression from the nmt81 promoter) to that of cells where transcription from the nmt81 promoter was repressed by addition of thiamine (while the fusion protein was "active"). In the latter case (transcriptional regulation), repression of the promoter still allowed enough Cdc13-des2-HDB expression to produce an anaphase delay (see shaded bar, left panel on Fig. 2B). In contrast, when the cells expressing the fusion protein were grown in the absence of estradiol (fusion protein "inactive"), the anaphase index corresponds to that of wild type cells that do not carry the *nmt.cdc13-des *construct indicating that the fusion protein is indeed inactive (see "HBD-E", right panel on Fig. 2B). We conclude that negatively regulating Cdc13-des2 protein activity using the HBD tag results in lower background activity than regulating transcription with the nmt promoter.

### Psf2-HBD

**Figure 3:**
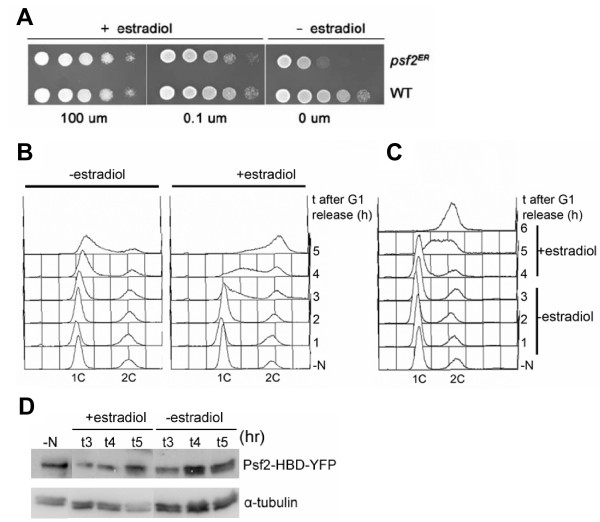
**Psf2-HBD is inactivated in the absence of estradiol**. **A**, *psf2-HBD *and wild type cells were serially diluted and spotted onto YE plates containing estradiol at the indicated concentrations. **B**, Strain P1520 (*psf2-HBD:kanMX*6) was grown in EMM plus 125 mM estradiol to log phase then shifted to EMM-N (+estradiol) for 16 at 25°C. Cells were released from the block by transferring to EMM+N in the absence (left) or presence (right) of estradiol. **C**, As in A, except that cells were released into EMM+N in the absence of estradiol, and 125 nM estradiol was added at 4 h. **D**, Cell extracts were made using the TCA method from cells incubated with and without estradiol as indicated. The extracts were run on protein gels and Psf2 was detected using an antibody against the YFP tag.

GINS is a tetrameric complex essential for the initiation and elongation steps of DNA replication [19,20]. The four subunits of GINS are essential for cell viability in budding yeast [19,20] therefore analysis of GINS function requires the isolation of conditional mutant alleles. In fission yeast temperature-sensitive alleles of the Psf2 and Psf3 subunits have been isolated and it was shown that Psf2 and Psf3 are required for DNA replication [21,22]. We explored whether the HBD could confer conditionality on the Psf2 subunit of fission yeast GINS. We fused sequences encoding the ERHDB to the 3' ends of the *psf2*^+ ^gene in the chromosome using the PCR-mediated gene targeting method [23]. Haploid cells expressing Psf2-ERHBD were viable when grown in the presence of estradiol in the growth medium but were inviable on medium lacking estradiol (Fig. 3A).

To determine whether the lethality was indeed due to a defect in DNA replication, a strain expressing Psf2-ERHBD was arrested in G1 by nitrogen starvation and released from the block in the presence or absence of estradiol. Cells released from the starvation block in the absence of estradiol ("inactive") only show some evidence of DNA replication at 5 h (Fig. 3B left panel), consistent with a role of Psf2 in DNA replication. Cells released from the starvation block in the presence of estradiol (fusion protein "active") carry out DNA replication 3–4 h after release (Fig 3B right panel) confirming that the fusion protein is indeed active. These data demonstrate that the ERHBD tag confers conditionality on Psf2 and the fusion protein can be activated by estradiol. Similar results were obtained with Psf1-ERHBD (data not shown, manuscript in preparation), the activity of which is also regulated by estradiol.

A similar experiment had been performed by Gomez et al [21] using the temperature sensitive *psf2 *allele, where the cells were arrested by nitrogen starvation and then released from the block at the restrictive temperature. It is interesting to note that the *psf2-HBD *allele arrests more tightly than the available *psf2*^*ts *^allele (compare fig. 5 in [21] to fig. 3B in the current paper). The mechanism of leakage at the late time-points in *psf2-HBD *is not known. Possible mechanism include release of some fusion protein from the Hsp90 complex even in the absence of estradiol or the fusion protein might be cleaved such that wild type Psf2 is produced.

We addressed the possibility that the presence of the tag might affect the stability of Psf2 and performed western blot analysis of extracts prepared from cells grown in the presence and absence of estradiol. Neither the N starvation-refeed procedure we used to synchronize the cells, nor the presence or absence of estradiol significantly affect the level of Psf2 (Fig. 3D).

To explore the reversibility of the arrest caused by inactivation of Psf2 -HBDby the absence of estradiol, cells were initially released from the N starvation block for 4 h in the absence of estradiol (fusion protein "inactive"). As shown above, the cells remain arrested with a 1C DNA content during this time (Fig 3C). After the 4-hour incubation in the absence of hormone, estradiol was added to the culture (fusion protein "active"). The cells carry out substantial DNA replication within 1 h, and replication is largely complete by 6 h (Fig. 3C) suggesting that the estradiol block is rapidly reversible.

### Limitations

#### HO-HBD

HO is an endonuclease that initiates mating-type switching by generating a double-strand break in the DNA in budding yeast [24,25]. Since the double-strand break occurs at a specific site, its fate can be conveniently investigated at the molecular level. Therefore, HO activity is often exploited to investigate checkpoint and repair pathways. However, such studies in fission yeast are hampered by the poor regulatability of the expression of HO. Its expression from the nmt promoter leads to a gradual accumulation of double strand breaks, which are processed as they arise. Thus, a mixed population of cells is investigated at any one time during the course of such an experiment, making it difficult to interpret the results. We therefore fused the ERHBD to the C-terminus of the HO endonuclease to test whether regulation of HO protein function by estradiol would provide a better tool to create double strand breaks in a controlled manner. We found that the HO-HBD fusion protein retains only a little endonuclease activity as compared to untagged HO, even in the presence of estradiol ("active") [see Additional files 1 and 2]. Similar result was obtained with an N terminally tagged HBD-HO fusion protein (Yari Fontebasso and Johanne Murray, personal communication).

#### Wee1-HBD

There are several protocols to synchronise *S. pombe *cells in different parts of the cell cycle. Induced synchronisation is often preferred over selection synchronisation because it is experimentally easier, especially for large cultures, and gives a high level of synchrony. However, induced synchronisation is usually dependent on temperature shifts which are sometimes not desirable. We sought to use Wee1 to generate synchronous cultures without involving a temperature shift. Wee1 is a protein kinase that inhibits entry into mitosis by phosphorylating Cdc2 [26,27]. Overexpressing Wee1 leads to a reversible G2 arrest. However, the currently available expression systems allow too strong expression even when *wee1 *is repressed, since fission yeast cells delay in G2 and become elongated even when one extra copy of *wee1 *is introduced into the cells. Therefore, long term overexpression can only be achieved if the overproduced Wee1 protein is inactive. We attempted to inactivate Wee1 by fusing it to the HBD. We fused the HBD to the C-terminus of Wee1, where the catalytic domain is located. We found that the Wee1-HBD fusion protein retains its activity in the absence of estradiol ("inactive") [see Additional files 1 and 3].

## Discussion

Here we show that the estradiol-regulatable hormone-binding domain provides a means to regulate efficiently and quickly the function of some fission yeast proteins, namely Cdc13-des2 and Psf2. In contrast, the HO-HBD fusion protein retains little activity even in the absence of estradiol ("active"), while the Wee1-HBD fusion protein was active even in the absence of estradiol ("inactive").

The Hsp90 complex was highly conserved through evolution. Therefore we expected that the HBD tag might confer sensitivity to estradiol to proteins in fission yeast. Analysis of each HBD-tagged protein requires an individual assay, therefore a large-scale analysis of the regulatability of fission yeast proteins is not feasible. Since here we show that the activities of some fission yeast proteins fused to the HBD are indeed regulated by estradiol, we speculate that the mechanism of regulation is probably through binding to Hsp90, as it is in other organisms.

In those cases when the ERHBD tag confers regulatability, the rate of activation and the tightness of the "off" state favourably compare with those obtainable with currently available expression systems. Fast activation of the fusion proteins is reflected in the rapid increase of the anaphase index and swift entry into S phase after activation of Cdc13-des2-HBD and Psf2-HBD, respectively. In the absence of estradiol (fusion protein "inactive") a tight inactivation is observed in both cases; cells expressing Cdc13-des2-HBD do not delay in anaphase and *psf2-HBD *cells remain arrested with unreplicated DNA for at least one generation time.

Switch-off experiments require removal of estradiol by extensive washing, which in itself stresses the cells and might be undesirable in a physiological experiment. However, as inactivation is tight and it does not require potentially time-consuming protein degradation once estradiol is removed, we expect that the system will be usable to switch off protein function within the time-scale of one cell cycle.

Since the initial discovery that HBD-tagged heterologous proteins are subject to hormonal regulation [13] a large number of proteins from various organisms has been tagged [28]. It is difficult to predict whether a fusion protein will be regulated by the hormone. In general, the effectiveness of the system may be determined by how the Hsp90 complex is positioned relative to the key functional domains of the tagged protein. We have fused the HBD close to the kinase domain of Wee1, expecting it to be inactivated by such a fusion. Apparently, the kinase domain might not be accessible to the Hsp90 complex, since the fusion protein is active in the absence of estradiol. However, Wee1 binds Hsp90 and this interaction protects it from degradation by the proteasome [29-31]. Although it is not clear which motifs or structural elements in protein kinases are recognized by the Hsp90 chaperone, the kinase domain is a possible candidate site of interaction. Thus, estradiol might not be able to induce a conformational change that is sufficient to override the interaction between Wee1 and Hsp90. Few endogenous Hsp90 substrates are known in fission yeast. Regulating such substrates with the HBD tag will obviously be difficult.

It is noteworthy that the proteins that were regulated by the HBD fusion and estradiol were proteins that depend on complex formation with other proteins for their function. Components of protein complexes might be more sensitive to regulation by steric interference, because complex formation may be affected. This conclusion is in line with the general trend observed in a large number of HBD fusion proteins [28]. It appears that proteins that must interact with other proteins or DNA to carry out their function, such as transcription factors or recombinases, have been successfully regulated by fusion to the HBD and estradiol presumably because their function can be inhibited by steric interference [12,13,28]. Simultaneous regulation of several components of a complex through this approach might be even more effective. On the other hand, enzymes such as β-galactosidase, galactokinase or URA3, that have small molecules as substrates, were not inactivated by steric interference by Hsp90 [13].

The classic model of steroid hormone receptor (SHR) action dictates that SHR-s are sequestered by chaperones in the cytoplasm and are released upon hormone addition. Indeed, Hsp90 is mainly cytoplasmic, but at least in some cell types it is also nuclear, especially after certain stresses [32-34]. In fission yeast Hsp90 is mainly cytoplasmic, but it is not excluded from the nucleus [35]. Localisation signals on the target protein might not be concealed by interaction with the chaperone, so different localisation signals might compete to determine the localisation of the "inactive" fusion protein. Thus, the intracellular localisation of a fusion protein in the absence of hormone is difficult to predict. After hormone addition, the localisation signals on the tagged protein are expected to determine the localisation of the fusion protein.

## Conclusion

The estradiol-regulatable hormone-binding domain provides a means to regulate efficiently and quickly the function of at least some fission yeast proteins. In some cases the system provides lower background protein activity and better kinetics of regulation than currently available regulatable expression systems. Since fission yeast is a useful model organism in a number of areas of biological research, this tool will greatly facilitate research in these fields.

## Methods

### General fission yeast methods

General fission yeast methods and growth media were as described before [36]. Estradiol (Sigma E2758) was made as a 10 mM stock in ethanol and used at a final concentration of 125–500 nM. Nourseothricin (ClonNAT) was obtained from Werner Bioagents. Cells were grown in EMM medium with supplements as required. Thiamine was made as a 10 mg/ml stock in water and used at a final concentration of 5 μg/ml in EMM. To derepress the *nmt *promoter, the cultures were washed three times with water and reinoculated at appropriate cell density in EMM.

### Plasmid and strain constructions

The strains used in this work are listed in Table 1.

**Table 1:** Strains used in this study

**Strain**	**Carrying the plasmid**
ade6-M210 leu1-32 h^-^	cdc13-des2-pREP41
	cdc13-des2-pREP81
ade6-M210 ura4-D18 h^+^	cdc13-des2-ERHBD-pREP81
	HO-ERHBD-pREP81
	wee1-ERHBD-pREP81
psf2-ERHBD:kanMX6 h^+^	
psf2-ERHBD:YFP:kanMX6 h^-^	

### pFA6-ERHBD-kanMX6

A C terminal tagging vector in the pFA6a-kanMX6 series [23] was constructed by replacing the GFP region with the HBD in pFA6a-GFP(S65T)-kanMX6 [23,37]. HBD was amplified from pHCA/GAL4(848).ER (D. Picard) as a PacI-AscI fragment using the following primers:

AAAA **TTA ATT AA**C TCT GCT GGA GAC ATG AGA GCT GCC

AAAA **GG CGC GCC**TCA GAC TGT GGC AGG GAA ACC CTC TGC and inserted into PacI AscI digested pFA6a-GFP(S65T)-kanMX6.

### Cdc13-des2-HBD, Wee1-HBD and HO-HBD

The *cdc13-des2, wee1 *and *HO *genes were amplified by PCR with Nde1 site introduced at START and Pac1 site introduced upstream of STOP using the primers shown in Table 2 (the sequences for the introduced sites are underlined): The PCR products were cut with Nde1 and Pac1.

**Table 2:** Primers used for plasmid construction

	Primers	Template
*cdc13-des2-HBD:*	TCCTC**CATATG**ACTACCCGT	pREP81-cdc13-des2
	A CAC TAA A**TT AAT T**AA CCA TTC	
*wee1-HBD*:	GGAATTC**CATATG**AGCTCTTCTTCTAATAC	Genomic
	C C**TT AAT TAA**AAC ATT CAC CTG CCA ATC TT	
*HO-HBD*:	GGAATTC**CATATG**CTTTCTGAAAACACGAC	Genomic
	C C**TT AAT TAA**GCA GAT GCG CGC ACC TGC GT	

HBD was isolated as a Pac1 – Asc1 fragment from pFA6-ERHBD-kanMX6. The pREP82 plasmid was cut with Sma1 and Asc1 linker was inserted. The Nde1 and Pac1 cut *cdc13-des2*, *wee1 *and *HO *PCR products were ligated with the HBD into Nde1 Asc1 cut pREP82-Asc1.

### Psf2-HBD

In order to tag the *psf2*^+ ^gene in the chromosome, the PCR-mediated gene targeting method for fission yeast [23] was used with plasmids pFA6a-HBD-kanMX6 and pFA6a-HBD-natMX6 as templates, the latter being constructed by transferring the PacI-AscI HBD fragment from the former into pFA6a-GST-natMX6 [38]. The primers used for amplification are shown below. Sequences with identity to the template plasmid are underlined.

PSF2-CTAG-5 5'-TGGAAATTAACGAAATACGTCCTATATTTCGAGAG GTGATGGACAGAATGCGCAAAATTGTTCAAGTTTCCCAAGAAGAACGGATCCCCGGGTTAATTAA-3'

PSF2-CTAG-3 5'-ATTTCACTACTACAAAGTTGGTATTCATAAACACTT CGTAGGATTCATTATCATTATTTTTAAAGTACATCATCCACACGGAATTCGAGCTCGTTTAAAC-3'

The resulting PCR products (5–10 μg) were transformed into S. pombe h^-N ^and h^+S ^strains as described and transformants selected with either 100 μg/ml G418 or 100 μg/ml nourseothricin [23,38]. Transformants were then screened by PCR to confirm that the gene was successfully tagged. The sequences of the PCR primers used for this can be obtained from the authors on request.

### Flow cytometry

Was performed using SYTOX Green as described previously [8].

### Immunoblots

Cell extracts were made by the TCA protein extraction method [39]. Detection was performed using the ECF or ECL kits (Amersham Biosciences).

## Authors' contributions

CAB made and characterized the HO-ERHBD construct under the guidance of BG. IG tagged the *psf2 *gene in the chromosome and performed the initial analysis of the tagged strain under the direction of SAM. CCP characterized the *psf-ERHBD *strain under the direction of SK. JS constructed the pFA6-ERHBD-kanMX6 plasmid under the guidance of MT. HCS constructed the *nmt82.cdc13-des2-ERHBD *plasmid and performed the initial characterization of the construct under the guidance of BG. EB, SK, SAM and MT contributed to writing the manuscript and designing experiments. BG constructed and characterized the *nmt82.wee1-ERHBD *plasmid, completed the characterization of *nmt82.cdc13-des2-ERHBD *and wrote the manuscript.

## Supplementary Material

Additional file 1 Limitations of the system. Two examples where fusing the ERHBD tag to the protein of interest did not lead to regulatability with estradiol are presented. Click here for file

Additional file 2 HO-HBD has little HO activity even in the presence of estradiol. The data provided present evidence that the HO-HBD fusion protein retains little endonuclease activity even in the presence of estradiol. Click here for file

Additional file 3 Wee1-HBD is active even in the absence of estradiol. The data provided present evidence that the Wee1-HBD fusion protein is active even in the absence of estradiol. Click here for file
