# Supplementary material for: Rapid regulation of protein activity in fission yeast
Source: BMC Cell Biol. 2008 May 5;9:23. doi: 10.1186/1471-2121-9-23 (PMC2408571; doi:10.1186/1471-2121-9-23)
Supplement: Additional file 1 — Limitations of the system. Two examples where fusing the ERHBD tag to the protein of interest did not lead to regulatability with estradiol are presented. [file 1471-2121-9-23-S1.pdf]

Additional file 1.

HO-HBD

HO is an endonuclease that initiates mating-type switching by generating a double strand break in the DNA in budding yeast [23,24]. Since the double strand break occurs at a specific site, its fate can be conveniently investigated at the molecular level. Therefore, HO activity is often exploited to investigate checkpoint and repair pathways. However, such studies in fission yeast are hampered by the poor regulatability of the expression of HO. Its expression from the nmt promoter leads to a gradual accumulation of double strand breaks, which are processed as they arise. Thus, a mixed population of cells is investigated at any one time during the course of such an experiment, making it difficult to interpret the results. We therefore fused the *ERHBD* to the C-terminus of the *HO* endonuclease to test whether regulation of HO protein function by estradiol would provide a better tool to create double strand breaks in a controlled manner. Expression of the fusion protein was driven by the nmt81 promoter. In order to test the activity and kinetics of activation of the fusion protein, we employed a strain carrying a minichromosome with a recognition sequence for HO in the *kanR* gene [40]. We followed HO activity by Southern blotting, using the *kanR* gene as a probe [40]. As a positive control, we used a strain carrying a plasmid with the wild type *HO* gene driven by the same promoter. A 6 kb fragment representing the uncut *kanR* gene was detected at all timepoints. Very little cutting was detected when the promoter was repressed or after 27 h of induction (Fig. 4 and not shown). After 30 h of induction the appearance of a 3.5 kb fragment indicated cutting in the control strain expressing wild type HO. However, we detected very little cutting in the strain expressing the HO-HBD fusion protein even in the presence of estradiol (fusion protein “active”) (Supplementary fig. 1). We conclude that the HO-HBD fusion protein retains only a little endonuclease activity as compared to untagged HO, even in the presence of estradiol (“active”).

Wee1-HBD

We sought to use Wee1 to generate synchronous cultures without involving a temperature shift. Wee1 is a protein kinase that inhibits entry into mitosis by phosphorylating Cdc2 [25,26]. Overexpressing Wee1 leads to a reversible G2 arrest. However, the currently available expression systems allow too strong expression even when *wee1* is repressed, since fission yeast cells delay in G2 and become elongated even when one extra copy of *wee1* is introduced into the cells. Therefore, long term overexpression can only be achieved if the overproduced Wee1 protein is inactive. A temperature-sensitive mutant has been successfully employed [41], but cell synchronisation required a temperature shift. We attempted to inactivate Wee1 by fusing it to the HBD. We expected that the fusion protein could be expressed to a high level in the absence of estradiol (fusion protein “inactive”) but it would mediate a G2 arrest after addition of estradiol (fusion protein “active”). Upon inactivating Wee1 by removal of estradiol the cells would enter a synchronous mitosis.

We fused the HBD to the C-terminus of Wee1, where the catalytic domain is located. Expression of the fusion protein was driven by the weak nmt promoter. We found that cells carrying the fusion protein grew poorly in the absence of estradiol (fusion protein “inactive”), even with the promoter repressed. They died as elongated cells with the promoter induced (Supplementary fig. 2). When estradiol was added to the cells, no dramatic difference was observed either in cell length or apparent generation time (data not shown). We conclude that the Wee1-HBD fusion protein retains its activity in the absence of estradiol (“inactive”).

Additional figures

Additional figure 1. HO-HBD has little HO activity even in the presence of estradiol.

Cutting in the *kanR* gene. A control plasmid (lane 1) or a plasmid carrying the wild type *HO* gene (lanes 2-5) or a plasmid carrying *HO-HBD* (lanes 6-10) was introduced into a strain carrying a minichromosome with the HO recognition sequence in the *kanR* gene. Lanes 2 and 6 show cutting in cells grown in the presence of thiamine for 30 h 15 min. The nmt81 promoter was induced for 30 h 15 min (lanes 3, 7, 8); 30 h 30 min (lanes 4, 9) and 31 h (lanes 5, 10). Estradiol was added after 30h (lanes 8, 9, 10; 15, 30 and 60 min with estradiol, respectively). The upper panel shows a southern blot using the *kanR* gene as a probe. The 6 kb band represents the uncut *kanR* gene, the 3.5 kb band represents the cut gene. The graph shows the relative intensity of the cut/uncut bands. The presence or absence of thiamine and estradiol, as well as the length of time in the absenece of thiamine and in the presence of estradiol, are indicated. na: not applicable

Additional figure 2. Wee1-HBD is active even in the absence of estradiol.

Cells were grown in the presence (**A**) and absence (**B**) of thiamine. Inserts show a wild type cell in anaphase for comparison. Bar represents 10μM.
