# Supplementary figures and images for: Rapid regulation of protein activity in fission yeast
Source: BMC Cell Biol. 2008 May 5;9:23. doi: 10.1186/1471-2121-9-23 (PMC2408571; doi:10.1186/1471-2121-9-23)

HO

HO-ERHBD

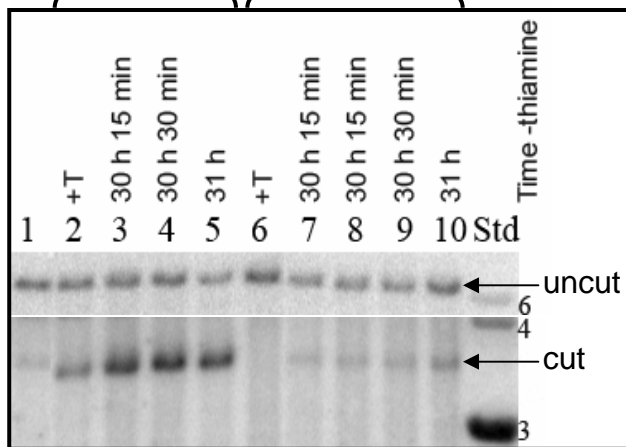

na + - - - + - - - Thiamine

na na na na na - - + + + Estradiol

15 30 60 Minutes in estradiol

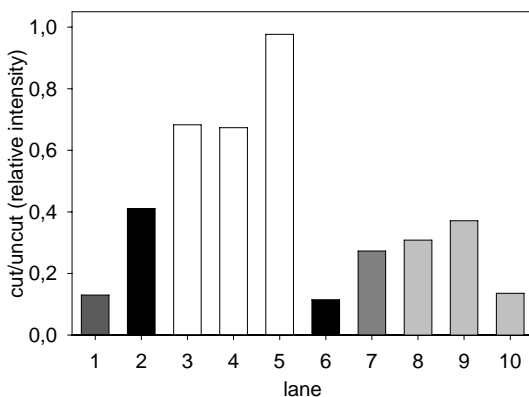

Supplement: Additional file 2 — HO-HBD has little HO activity even in the presence of estradiol. The data provided present evidence that the HO-HBD fusion protein retains little endonuclease activity even in the presence of estradiol. [file 1471-2121-9-23-S2.pdf]

A

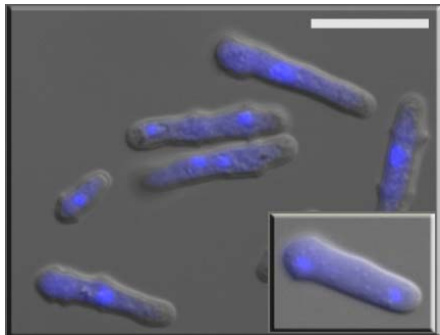

B

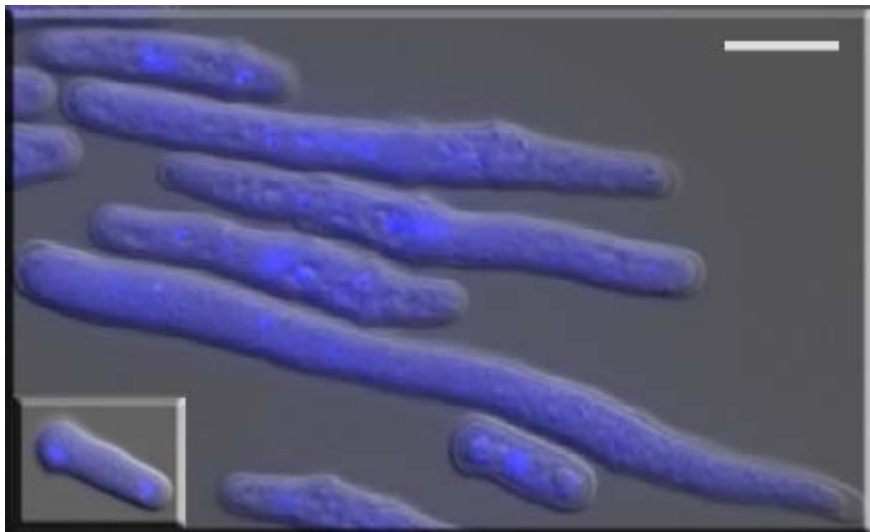

Supplement: Additional file 3 — Wee1-HBD is active even in the absence of estradiol. The data provided present evidence that the Wee1-HBD fusion protein is active even in the absence of estradiol. [file 1471-2121-9-23-S3.pdf]
